# Effects of dietary capsaicin supplementation on growth performance, blood profile and carcass and meat quality of finishing pigs

**DOI:** 10.5713/ab.23.0541

**Published:** 2024-05-07

**Authors:** Siriporn Namted, Kanokporn Poungpong, Chaiyapoom Bunchasak

**Affiliations:** 1 Department of Agriculture, Faculty of Agriculture Technology, Valaya Alongkorn Rajabhat University Under the Royal Patronage, Pathum Thani Province 13180, Thailand; 2 Department of Animal Science, Faculty of Agriculture, Kasetsart University, Bangkok, 10900, Thailand

**Keywords:** Blood Profile, Capsaicin, Growth Performance, Meat Quality, Pigs

## Abstract

**Objective:**

This study was conducted to determine the effects of capsaicin (CAP) on productive performance, blood profile, intestinal morphology, carcass and meat quality of growing-finishing pigs.

**Methods:**

Two experimental diets were offered to 36 crossbred barrows: basal diet (0% CAP) and basal diet with CAP at 0.02%. Each experimental group consisted of 18 pigs, with six replications (three each).

**Results:**

Supplementation of CAP at 0.02% decreased average daily feed intake (p = 0.003) and feed cost/gain (p = 0.056), increased return on investment (p = 0.052) and increased gain:feed ratio (p = 0.037) during the growing period. There was no effect of CAP on the growth rate. The blood urea nitrogen and nitrogen (N) levels in faeces tended to decrease (p = 0.093 and p = 0.087), whereas the basophil level increased with CAP supplementation (p = 0.029). In addition, dietary CAP supplementation decreased crypt depth (p = 0.022) and tended to increase the villus height/crypt depth ratio in the segment of the jejunum (p = 0.084). Backfat (BF) thickness (p = 0.047) was reduced by supplementing CAP. Whereas the protein content increased with CAP supplementation (p = 0.021). Using CAP in the diet of growing pigs increased the pH at 6 h post-mortem (p = 0.046) and tended to increase the springiness value (p = 0.078) of the meat. In terms of meat color, CAP supplementation increased the yellowness (p = 0.029).

**Conclusion:**

Supplemental CAP improves gut morphology and blood profiles, consequently promoting productive performance as well as carcass and meat quality.

## INTRODUCTION

Chilli extracts (capsaicin; CAP) are being used in pig diets due to several benefits, such as increasing nutrient digestion and absorption, reducing fat accumulation and pathogenic bacteria, increasing anti-oxidant properties as well as enhancing immune functions and anti-inflammatory activity [1]. From 2009 to 2019, world production of the chilli was increased by 10 million tonnes per year, from 28 to 38 million tonnes [2]. Particularly, the chilli is an important crop in Thailand that grows around 100,000 hectares in all parts of the country [3].

The CAP (trans-8-methyl-N-vanillyl-6-nonenamide) is a compound of lipophilic alkaloid [4], and up to 80% is passively absorbed in the segments of the stomach and the duodenum [5]. Several authors reported that the addition of CAP to pig diets significantly improved the small intestinal morphology of piglets [6] and increased the secretion of digestive enzymes through neurostimulatory activity [7]. According to other studies, CAP binds to heat-sensing receptors and potentially reduces heat stress by manipulating cellular metabolism [1,7], providing antioxidant activity [8], and improving passive immunity [9].

However, there are few studies on the effects of using CAP in the diet of fattening pigs on carcass and meat quality. In this context, the objective of the present study was to assess the effect of adding CAP to the diet of pigs from the grower to the finisher period on growth performance, blood profile, small intestinal morphology, carcass characteristics and meat quality.

## MATERIALS AND METHODS

The animal welfare procedures were approved by the Kasetsart University Institutional Animal Care and Use Committee (ID: ACKU66-AGR-019). The animals were handled (license number U1-07385-2561) at the Animal Research Farm (NKP Farm), Rayong Province, Thailand.

### Animals and management

According to a completely randomized design, 36 castrated pigs (Large White×Landrace×Duroc) with an average body weight (BW) of 35±0.1 kg were used. The pigs were divided into two experimental groups. Each experimental group consisted of six replications and three pigs each. The pigs were kept in pens (1.0×1.2 m^2^) with an evaporative cooling system. The average temperature in the house was 29°C, and water and feed were provided *ad libitum*.

### Experimental diets

Diets were provided in two phases (growing period; 35 to 60 kg and finishing period; 60 to 100 kg). The experimental diets were formulated according to the guidelines of the NRC [10]. Calculations for nutrient density and some nutrient analyses are shown in Table 1. The basal diet was assigned to the control group, and the CAP was added to the basal diet at an inclusion level of 0.02% (20 mg/kg; treatment group).

### Growth performance

The body weigh at 35, 60, and 100 kg was measured. Average daily feed intake (ADFI) and BW gain were recorded, and the gain:feed, feed cost/gain (FCG) and return on investment (ROI) were determined. At the end of the study, six pigs that had a BW close to the average BW of the group were chosen from each experimental group, and the feed was withdrawn for 12 h.


$$\begin{array}{l} \text{FCG}=(\text{Feed cost }1\;\text{kg}\times \text{Feed intake})/\text{BW gain}\\ \text{ROI}=[([\text{BW gain}\times \text{Pork price}]-[\text{Feed cost }1\;\text{kg}\times \text{Feed intake}])/(\text{Feed cost }1\;\text{kg}\times \text{Feed intake})]\times 100 \end{array}$$

### Blood analysis

At the end of the trial, 6 pigs from 2 replicates were selected (BW close to the mean for the group). Subsequently, blood was taken from the jugular vein, and blood urea nitrogen (BUN), white blood cell (WBC) count and total immunoglobulin G (IgG) were determined. Serum samples were isolated (centrifuged at 3,000×g for 15 min) 2 to 4 h after collection. The BUN level was analysed using a commercial strip test (Urea liquicolor No.10505; Human Diagnostics Worldwide, Wiesbaden, Hessen, Germany). Triplicate glass slides were prepared for blood films and stained with Giemsa-Wright stain. White blood cells (neutrophils, lymphocytes, monocytes, eosinophils and basophils) were counted under a microscope according to Borges et al [11]. The total IgG in the blood was assayed using the radial immunodiffusion technique (Bethyl Laboratories, Inc., Montgomery, TX, USA).

### Morphology of the small intestine

After blood collection, each pig was slaughtered by electrical stunning on d 163, according to standard commercial procedures. Tissue samples from the duodenum, jejunum and ileum were taken, fixed in 10% buffer formalin and placed in paraffin. For each specimen, according to Sakdee et al [12], haematoxylin-eosin was used for staining the tissue (at least 10 sections of 7-μm thickness) to evaluate the histological parameters under a light microscope. The morphology of each small intestinal tract (villous height, crypt depth and villous height/crypt depth ratio) was determined by a computer-assisted image-analysis (Biowizard, Thaitec, Thailand).

### Carcass measurements

Within 1 hour post-mortem, each hot carcass weight (without the head) was determined for the calculation of the percentage of the dressing carcass. Carcass length (cm) was determined from the cranial edge of the 1st rib to the cranial tip of the aitch bone [13]. The average backfat (BF) and fat-lean ratio values of 12 carcasses were measured. Digital vernier calipers were used to determine the BF thickness at five positions, namely the 1st rib (BF1), the 13th rib (BF2), the front base of the Gluteus Medius (BF3), the thinnest part of the BF (BF4) and on the top of the Gluteus Medius (BF5) (Fowler High Precision, Newton, MA, USA). The width of the gluteus muscle was also measured (b).

According to Chaweewan et al [14], the percentage of lean muscle and fat free index were estimated. The average BF and lean muscle ratio were evaluated using the Lenden-Speck-quotient (LSQ) index according to the equations of Lothong et al [15]:


$$\begin{array}{l} \text{LSQ}=(\text{BF}3+\text{BF}4)/(2\text{b})\\ \text{Average BF}=[\text{BF}1+\text{BF}2+(\text{BF}3+\text{BF}4+\text{BF}5)/3]3\\ \text{Percentage of lean}=49.123-0.55983\text{BF}4+0.22096\text{b}\\ \text{Fat free index}=50.767+(0.035\times \text{hot carcass weight in kilogram})\\ -(8.979\times \text{last rib midline back-fat on hot carcass in centimetre}) \end{array}$$

### Meat quality measurements

The pH of the longissimus muscle between the 13th and 14th rib on the intact carcass was determined at 45 min, 6 h and 24 h post-mortem by a portable pH meter (Instruments, Wilmington, MA, USA) with a puncture electrode. At the 10th rib and the P2 position, BF thickness was determined using a 0.5-mm flexible ruler (Mitutoyo, Andover, Hampshire, UK) [14].

#### Sample sectioning

The surface color of the longissimus muscle at the 13th thoracic segment was measured at 45 min post-mortem, and the sample was stored at −20°C. Drip loss, cooking loss and proximate composition were analysed according to Davila-Ramirez et al [16].

#### Meat color

The parameters L* (lightness), a* (redness) and b* (yellowness) were used to indicate the color, employing a Minolta CR-400 colorimeter (Konica Minolta Sensing, Inc., Tokyo, Japan). The overall color change (ΔE) was calculated as follows:


$$\Delta \text{E}={\left[{\left(\Delta {\text{L}}^{*}\right)}^{2}+{\left(\Delta {\text{a}}^{*}\right)}^{2}+{\left(\Delta {\text{b}}^{*}\right)}^{2}\right]}^{1/2}$$

where ΔL*, Δa*, and Δb* are the differences between the control and the CAP group values for L*, a*, and b*, respectively. The hue angle (Hue) was calculated using the formula Hue = tan^−1^ (b*/a*), and chroma was calculated using the formula chroma = (a*+b*)^1⁄2^ [16].

#### Drip loss

To determine the drip loss, the muscle was thawed for 24 h at 4°C. Using the plastic-bag method, the drip loss at 24 h, based on the equation of Honikel [17], was calculated:


Drip loss=[(Initial fresh meat weight-Meat weight after chilling)×100]/Initial fresh meat weight

#### Cooking loss

The weight of the raw longissimus muscle (14th thoracic vertebra) was determined, and the muscle was heated immediately in a thermostat water bath (HH-4, Jiangbo Instrument, Jiangsu, China) up to 71°C. Subsequently, the cooked meat was cooled down to room temperature (25°C) and weighed to evaluate the cooking loss [16].


Cooking loss=[(Initial fresh meat weight-Meat after cooking)×100]/Initial fresh meat weight

#### Texture analysis

The firmness of the longissimus muscle was measured via the Warner-Bratzler shear force (WBS). For this, cooked meat squares with the dimensions of 1×1×2.5 cm (height×width×length) (from cooking loss determination) were used [16].

Texture profile analysis (TPA) was performed in a TA-XT texture analyser (Stable Micro System, Godalming, Surrey, UK) prepared with a TA-7 Warner-Bratzler blade. The cooked meat was shaped into a cylinder (30 mm height × 30 mm diameter) and examined with two compression cycles; subsequently, the force was outlined on a force-time plot. The data were used to calculate hardness, cohesiveness, springiness, gumminess and chewiness [18].

#### Chemical meat composition

Each longissimus muscle at the 14th thoracic vertebra was triplicated and analysed for the water (at 100°C for 16 h; method 950.46), crude protein (Kjeldahl method 955.04) and crude fat (Folch and Soxhlet methods 920.39) contents, according to the AOAC [19].

#### Fecal nitrogen content

To determine the nitrogen (N) content of the, 100 g of from each treatment was randomly collected and stored at −20°C. Subsequently, the were thawed and dried at 60°C±2°C for 24 h, and the N concentration in the dry was determined using the Kjeldahl method (method 955.04) [19].

### Statistical analysis

The pen was defined as the experimental unit. Analysis of covariance (ANCOVA) was performed to test the significant effect between 2 experimental groups.


$${\text{Y}}_{\text{ij}}=\mu +\tau_{\text{i}}+\beta ({\text{x}}_{\text{ij}}+\bar{\text{X}})+{ɛ}_{\text{ij}}$$

When; Y_ij_ is the observed response, μ is overall mean, τ_i_ is the effect of adding CAP in diet, β is regression value, x_ij_ is covariate value, X̄ is mean of x, and ɛ_ij_ is the experimental error.

A Student’s *t*-test was used to compare the values obtained from the two independent groups, including growth performance, blood profiles, intestinal morphology, carcass quality and meat quality, employing the Statistical Analysis System (SAS) Version SAS 9.1 software (SAS Inst. Inc., Cary, NC, USA). Data are expressed as mean with standard error of the mean. A value of p<0.05 was considered significant, and 0.05 ≤p<0.10 was defined as a tendency.

## RESULTS AND DISCUSSION

### Growth performance

Table 2 shows the effects of using CAP on productive performance. Using ACOVA, there was no effect of initial BW between the control and CAP groups on BW and gain:feed throughout the experimental period. At a BW of 35 to 60 kg, supplementing CAP decreased ADFI (p = 0.003), increased gain:feed (p = 0.037), decreased FCG (p = 0.056) and increased the ROI value (p = 0.052), but there were no effects of dietary CAP on these parameters during the finishing period (60- to 100-kg BW). For a BW of 35 to 100 kg, the ADFI tended to decrease (p = 0.075), and the gain:feed was increased (p = 0.073) by CAP supplementation.

Although there was no effect of CAP on the growth rate, supplementing CAP (20 mg/kg diet) increased feed efficiency (gain:feed) via a reduction in feed intake during the growing period, which may be due to an increase of digestion, absorption or both. The positive effect of CAP during this period may be due to higher feed utilization compared to the finisher period, because Sobolewska and Grela [20] found that the feed conversion ratio of grower period was better than that of the finisher period. Accordingly, Rosa-Medina [7] found that dietary CAP increases the levels of digestive enzymes (amylase, protease and lipase) and digestibility of feed. Moreover, CAP reduces the demand of energy for maintenance because CAP activates the transient receptor potential vanilloid 1 (TRPV1) receptor in the nervous system and reduces metabolic stress [21].

### Blood profiles and composition

Table 3 shows the effects of using CAP on the BUN, WBC, IgG, and N contents of the. The basophil (p = 0.029) concentration in the blood was increased by CAP supplementation, whereas the BUN and N concentrations in the feces tended to decrease (p = 0.093 and p = 0.087).

Supplementing CAP in the diet reduced the concentrations of BUN (−18%), and N in the feces (−5%) of growing-fattening pigs. In previous studies, CAP supplementation reduced the levels of BUN in pigs [6]. In general, a high blood urea concentration indicates a high catabolism of protein and metabolic stress [22]. It is assumed that adding CAP to pig diets during the growing period improve protein utilization and reduce stress of the pig because the gain:feed was improved via the reduction of feed intake.

The concentration of basophils in the serum increased by 27% under CAP supplementation compared with the control group. A basophil is a type of WBC that plays a role in the immune system, although it only accounts for approximately 1% or less of all WBCs. Basophils act against infection and are related to inflammation and allergy processes [23]. However, in present study, the mechanism of CAP on the elevation of basophils is not known. Akimoto et al [24] reported that CAP supplementation decreased the number of acquired immunity cells and increased the number of total WBCs by the different mechanisms from catecholamine. In addition, when sows were fed gestation and lactation diets containing 10 mg/kg of CAP, their piglets showed increased IgG concentrations in the serum until 28 d of age [9].

### Morphology of the small intestine

Table 4 shows the effects of adding CAP on small intestinal morphology (villous height, crypt depth, and villous height/crypt depth ratio). Supplementing CAP decreased jejunum crypt depth (p = 0.022) and tended to increase the jejunal villus height/crypt depth ratio (p = 0.084), whereas the other parameters were not affected by CAP supplementation.

The changes in the intestinal morphology following CAP supplementation may benefit the digestive tract [25]. The addition of CAP to piglet diets improved the small intestinal morphology [6]. This may be due to the property of antioxidation of the CAP [8]. Accordingly, the present study shows that crypt depth in the jejunum segment was lower, and the villi height/crypt depth ratio tended to be higher in the CAP group than the control. The jejunum is the main nutrient absorption area, and Markovi et al [26] indicated that the improvement in jejunal morphology is positively related to animal growth performance. In the present study, the villus height-to-crypt depth ratio was greater in the CAP group (approximately 40%), which may impact nutrient absorption and digestion. Consequently, the feed efficiency of the pigs was improved. Although a greater villus height is related to high brush border enzyme expression and improved nutrient transportation [27], the ratio of villus height to crypt depth value is also an important indicator of gut function.

### Carcass quality

Table 5 shows the effects of dietary CAP on the carcass compositions. Back fat (BF) thickness was decreased (p = 0.047), whereas carcass length tended to increase (p = 0.061) following CAP supplementation. There was a tendency for increased fat free index (p = 0.092) and lean percentages (p = 0.074) in meat from animals supplemented with CAP.

We observed losses of BF (−18%) and fat percentage in meat (−14%) due to CAP supplementation. In a similar study, a phytogenic mixture (capsicum, carvacrol, cinnamaldehyde) did not affect the BF of lactating sows [28]. Recently, Hui et al [29] stated that the intake of CAP affects the insulin levels in humans and animals. Insulin sensitivity and the expression of glucagon-like peptide-1 and insulin-like growth factor 1 induce glucose and amino acid transportation through blood circulation [30]. Moreover, Li et al [31] showed that dietary CAP stimulates the proliferator-activated receptor δ by TRPV1 activation, resulting in elevated autophagy and lipolysis. Elmas and Gezer [32] confirmed that CAP can increase lipolysis in white adipose tissue. Moreover, CAP increases energy expenditure through brown adipose tissue in healthy humans and animals through the central thermogenic neurons [33]. Recently, Kroscher et al [34] reported that CAP reduces the accumulation of fat by controlling the synthesis of bile acids, improving the gut microbiome and increasing the amounts of short-chain fatty acids. This leads us to infer that using CAP in diets improves energy and protein use and reduces fat accumulation through various mechanisms.

### Meat quality

Table 6 shows the effect of dietary CAP on the pH, color, water-holding capacity (WHC) and texture (WBS and TPA) of the meat. The muscle pH at 6 h post-mortem was higher in the CAP group (p = 0.046) compared to the control group. Meat yellowness (b*) at 45 min was lower in the CAP group (p = 0.029). Supplementing CAP increased the springiness value (p = 0.078). The crude fat level, WHC, WBS and pork color at 24 h were not affected by CAP supplementation, whereas CAP increased the meat protein content (p = 0.021).

The meat quality characteristics (pH, color, WHC, protein, texture, taste and shelf life) are influenced by genetic, dietary and environmental factors [35]. In the present study, the pH at 6 h was higher in the CAP group compared to the control group, with potential positive impacts on the shelf life of the pork [36]. Li et al [4] also found that adding 2 mg/kg CAP maintained the muscle pH at 24 h. The highest rate of glycogen degradation in pork is around 5 to 6 h post-mortem [36]. However, in the present study, dietary CAP supplementation maintained the level of pH at 6 h (normal range; 6.03>pH, at 6 h); a normal pH level prolongs the quality and shelf life of pork [37].

Meat color can indicate physiological, biochemical and microbiological changes in meat [4]. Dietary CAP supplementation reduced the b* value (yellowness; p = 0.029). This finding is in agreement with Pugliese et al [38], who reported that a more yellow (greater b*) meat is a result of a higher intramuscular fat content. Han et al [39] reported that total color differences for ΔE >0.6 were visually noticeable, whereas the ΔE in the pork muscle in the present study was 1.12. Thus, supplementing pig diets with CAP positively affected the color of the pork.

The WHC can be measured by the values of drip loss and cooking loss, and the tenderness can be indicated by the WBS value. In this study, the drip loss, cooking loss and shearing force of the pork were not affected by CAP supplementation. Similarly, in broiler chicken meat, Li et al [4] found no effect of dietary CAP supplementation on these values. The mechanical properties (TPA) such as hardness, springiness, chewiness, cohesiveness and adhesiveness are widely used to estimate the meat texture and can be applied to predict pork tenderness [40]. However, in this study, CAP supplementation did not influence the TPA values, although springiness was increased. The high springiness value normally increases the shear force of the pork. This phenomenon was probably due to the increase of protein concentration in the pork by CAP supplementation. Nitrogen content in pork was increased by 5.23%, while nitrogen content in feces and BUN was reduced by 8.62% and 18.30%, respectively. It can be hypothesized that CAP may increase both protein digestion and amino acids uptake form blood vessel to muscle cell.

## CONCLUSION

In conclusion, CAP supplementation reduced feed consumption during the growing period while maintaining the growth rate. Consequently, the gain:feed and the FCG were improved. These phenomena may be caused by the improvement of the jejunal morphology and blood profiles. Fat accumulation in the carcass decreased, whereas the protein content and the pH (at 6 h) of the meat increased. In this sense, using CAP in pig diets (growing-finishing state) improves productive performance and carcass or meat quality.

## Figures and Tables

**Table 1:** Ingredient and chemical composition of experimental diets

Items	Grower	Finisher
Ingredient (%)		
Broken rice	54.96	25.00
Rice bran oil	1.79	2.45
Corn	-	26.03
Soybean meal, 48% crude protein	24.67	23.89
Cassava	10.00	15.00
Rice bran solvent meal	4.91	5.00
L-Lysine hydrochloride, 78%	0.27	0.05
DL-Methionine	0.08	-
L-Threonine	0.11	-
Monodicalcium phosphate, 22% phosphorus	0.95	0.54
Limestone	0.94	0.79
Salt	0.32	0.25
Vitamin-mineral premix^1)^	0.25	0.25
Sodium bicarbonate	0.50	0.48
Antioxidant and toxin binder	0.25	0.25
Total	100.00	100.00
Chemical composition		
Metabolizable energy for swine (kcal/kg)	3,300	3,300
Crude protein (%)	17.0 (17.4)^2)^	16.0 (16.7)
Fat (%)	3.0 (3.0)	4.5 (4.9)
Fiber (%)	3.02	3.31
Standardized ileal digestible basis (%)		
Lysine	0.85	0.73
Methionine	0.24	0.21
Methionine+cysteine	0.48	0.42
Threonine	0.52	0.46
Tryptophan	0.15	0.13
Total basis (%)		
Lysine	1.12	0.88
Methionine	0.36	0.26
Methionine+cysteine	0.36	0.54
Threonine	0.72	0.61
Tryptophan	0.25	0.19
Calcium	0.65	0.52
Total phosphorus	0.66	0.49
Dietary electrolyte balance (mEq)	232	240

1) Vitamin and mineral premix contents; vitamin A (retinyl acetate) 4 MIU, vitamin D (cholecalciferal) 0.64 MIU, vitamin E (DL-α-tocopheryl acetate) 24,000 IU, vitamin K_3_ (menadione) 1.4 g, vitamin B_1_ (thiamine) 0.6 g, vitamin B_2_ (riboflavin) 0.3 g, vitamin B_6_ (pyridoxine) 0.75 g, vitamin B_12_ (cyanocobalamin) 14 mg, nicotinic acid 20 g, pantothenic acid 10 g, folic acid 0.44 g, d-biotin 0.04 g, choline chloride 60 g, Fe (FeSO_4_·H_2_O) 45 g, Cu (CuSO_4_·5H_2_O) 40 g, Mn (MnO) 15 g, Zn (ZnO) 40 g, Co (CoCO_3_) 0.2 g, [Ca(IO_3_)_2_] 0.4 g, Se (Na_2_SeO_3_) 0.06 g, carrier (ground corn cobb) added to 1 kg.

2) Values in parentheses are proximate values.

**Table 2:** Effects of supplementing capsaicin on the growth performance of pigs during the growing and finishing periods

Items	Control	Capsaicin	SEM	p-value
Initial weight (kg)	35.6	36.1	0.57	0.688
Grower pigs				
Body weight (kg)	65.3	65.6	0.64	0.824
Body weight gain (kg)	29.7	29.5	0.52	0.868
Average daily gain (g/d)	1,061	1,054	18.46	0.870
Average daily feed intake (g/d)	2,306	2,064	48.79	0.003
Gain:feed	0.462	0.514	0.013	0.037
Feed cost/gain (bath)	31.4	28.7	0.73	0.056
Return on investment	123.1	145.7	5.98	0.052
Finisher pigs				
Body weight (kg)	108.3	108.7	0.97	0.862
Body weight gain (kg)	43.0	43.1	0.61	0.967
Average daily gain (g/d)	1,102	1,103	15.67	0.966
Average daily feed intake (g/d)	2,881	2,832	41.67	0.588
Gain:feed	0.382	0.392	0.003	0.225
Feed cost/gain (bath)	33.8	33.6	0.33	0.758
Return on investment	107.2	108.8	2.04	0.719
Overall				
Body weight (kg)	108.3	108.7	0.97	0.862
Body weight gain (kg)	72.7	72.6	0.92	0.948
Average daily gain (g/d)	1,085	1,083	13.69	0.948
Average daily feed intake (g/d)	2,641	2,511	36.92	0.075
Gain:feed	0.410	0.432	0.006	0.073
Feed cost/gain (bath)	32.8	31.6	0.43	0.168
Return on investment	113.4	122.3	3.06	0.159

SEM, standard error of the mean. n = 6.

**Table 3:** Effects of supplementing capsaicin on blood parameters and feces of pigs

Items	Control	Capsaicin	SEM	p-value
Blood				
Blood urea nitrogen (mg/dL)	20.10	16.42	0.01	0.093
White blood cells (10^3^/μL)	21.15	21.66	0.51	0.730
Neutrophil (%)	27.82	24.36	0.22	0.570
Lymphocyte (%)	65.04	67.46	0.43	0.719
Neutrophil/Lymphocyte ratio	0.46	0.38	0.20	0.581
Monocyte (%)	4.08	3.36	0.12	0.379
Eosinophil (%)	2.68	4.30	0.02	0.200
Basophil (%)	0.38	0.52	0.02	0.029
Immunoglobulin G (mg/mL)	563.50	614.40	0.05	0.541
Feces				
Nitrogen content (%)	4.06	3.71	0.11	0.087

SEM, standard error of the mean. n = 6.

**Table 4:** Effects of supplementing capsaicin on the morphology of the small intestine

Items	Control	Capsaicin	SEM	p-value
Villus height (μm)				
Duodenum	270.76	311.51	18.81	0.313
Jejunum	258.90	275.77	14.85	0.600
Ileum	229.94	267.67	12.98	0.156
Crypt depth (μm)				
Duodenum	491.98	538.88	36.41	0.561
Jejunum	392.27	320.74	16.84	0.022
Ileum	293.50	341.42	21.68	0.295
Villus height to crypt depth ratio				
Duodenum	0.58	0.57	0.04	0.979
Jejunum	0.65	0.89	0.06	0.084
Ileum	0.81	0.79	0.04	0.811

SEM, standard error of the mean. n = 6.

**Table 5:** Effects of supplementing capsaicin on carcass quality of pigs

Items	Control	Capsaicin	SEM	p-value
Live weight (kg)	106.4	110.6	3.1	0.538
Carcass length (cm)	74.4	78.8	1.2	0.061
Carcass weight (kg)	83.6	85.7	2.2	0.670
Dressing percentage (%)	78.6	77.5	0.5	0.267
Backfat thickness (mm)	33.8	27.6	1.6	0.047
Lenden-Speck-quotient	0.40	0.29	0.03	0.111
Lean (%)	42.5	45.7	0.9	0.074
Fat free index	25.6	35.7	0.03	0.092

SEM, standard error of the mean. n = 6.

**Table 6:** Effects of capsaicin supplementation on meat quality and chemical composition

Items	Control	Capsaicin	SEM	p-value
pH				
pH at 45 min	6.51	6.52	0.07	0.953
pH at 6 h	6.14	6.45	0.08	0.046
pH at 24 h	5.71	5.64	0.06	0.624
Color at 45 min				
Lightness (L*)	48.72	48.51	0.16	0.576
Redness index (a*)	3.26	2.86	0.26	0.473
Yellowness index (b*)	4.64	4.25	0.09	0.029
Hue angle (H*)	55.40	56.72	1.93	0.751
Chroma (C*)	6.01	6.83	0.20	0.214
Overall color change (ΔE)	-	1.12	-	-
Water-holding capacity				
Drip loss (%)	2.38	2.18	0.23	0.694
Cooking loss (%)	26.52	27.94	0.52	0.198
Texture				
Shear force (kg)	80.68	98.85	12.43	0.498
Texture profile analysis				
Hardness (g)	620.80	764.16	71.41	0.344
Springiness	0.75	0.79	0.01	0.078
Cohesiveness	0.59	0.61	0.01	0.369
Gumminess	372.42	472.36	42.94	0.268
Chewiness	286.13	377.35	35.57	0.218
Chemical composition (% of DM)				
Moisture (%)	70.03	70.05	0.19	0.951
Dry matter (%)				
Nitrogen	3.82	4.02	0.30	0.072
Protein	23.87	25.16	0.30	0.021
Crude fat	9.85	7.79	0.98	0.321

SEM, standard error of the mean. n = 6.
